# Oligonucleotide Therapeutics as a New Class of Drugs for Malignant Brain Tumors: Targeting mRNAs, Regulatory RNAs, Mutations, Combinations, and Beyond

**DOI:** 10.1007/s13311-018-00702-3

**Published:** 2019-01-14

**Authors:** Anna M. Krichevsky, Erik J. Uhlmann

**Affiliations:** Ann Romney Center for Neurologic Diseases, Department of Neurology, Brigham and Women’s Hospital and Harvard Medical School, Initiative for RNA Medicine, Boston, Massachusetts 02115 USA

**Keywords:** brain tumors, microRNA, long noncoding RNA, mutations, epigenetics, gene editing.

## Abstract

Malignant brain tumors are rapidly progressive and often fatal owing to resistance to therapies and based on their complex biology, heterogeneity, and isolation from systemic circulation. Glioblastoma is the most common and most aggressive primary brain tumor, has high mortality, and affects both children and adults. Despite significant advances in understanding the pathology, multiple clinical trials employing various treatment strategies have failed. With much expanded knowledge of the GBM genome, epigenome, and transcriptome, the field of neuro-oncology is getting closer to achieve breakthrough-targeted molecular therapies. Current developments of oligonucleotide chemistries for CNS applications make this new class of drugs very attractive for targeting molecular pathways dysregulated in brain tumors and are anticipated to vastly expand the spectrum of currently targetable molecules. In this chapter, we will overview the molecular landscape of malignant gliomas and explore the most prominent molecular targets (mRNAs, miRNAs, lncRNAs, and genomic mutations) that provide opportunities for the development of oligonucleotide therapeutics for this class of neurologic diseases. Because malignant brain tumors focally disrupt the blood–brain barrier, this class of diseases might be also more susceptible to systemic treatments with oligonucleotides than other neurologic disorders and, thus, present an entry point for the oligonucleotide therapeutics to the CNS. Nevertheless, delivery of oligonucleotides remains a crucial part of the treatment strategy. Finally, synthetic gRNAs guiding CRISPR–Cas9 editing technologies have a tremendous potential to further expand the applications of oligonucleotide therapeutics and take them beyond RNA targeting.

## Cell and Molecular Landscape of Malignant Gliomas

### Complexity of Malignant Glioma and Therapeutic Challenges

Glioblastoma (GBM, or astrocytoma WHO grade IV) is diagnosed at a rate of 3 to 4 new cases per 100,000 individuals in the developed countries, corresponding to about 13,000 new cases annually in the USA [1–3]. Despite aggressive treatment with maximum resection followed by concurrent radiotherapy and chemotherapy, the prognosis is still very poor. Merely four drugs and one device received the FDA approval for high-grade gliomas in 30 years, in spite of numerous preclinical and clinical studies. The alkylating agent temozolomide (TMZ) has efficacy both as single agent and with concurrent radiation and is standard-of-care. Median survival of patients receiving standard-of-care treatment remains around 15 months and less than 10% of the patients survive over 5 years. Therefore, new approaches and therapeutic strategies are critically needed to combat the disease. Extensive multidisciplinary research in neuro-oncology (mostly focusing on GBM) has prospered in the last decade and resulted in about 20,000 peer-reviewed publications. The growing body of knowledge will inevitably lead to therapeutic breakthroughs. The biological and clinical characteristics of GBM have been previously reviewed [1–3]. In the sections below, we will provide a brief introduction to the cell and molecular biology of GBM as a foundation for the development of oligonucleotide therapeutics (OT).

GBM is a highly complex tumor that consists of a tumor core and a surrounding ill-defined invasive zone. At the cellular level, bona fide glioma cells contribute to ~ 80% of the tumor mass. The rest comprise of endothelial cells and blood vessels along with the macrophages, microglia, myeloid cells, lymphocytes, and other infiltrating immune cells that are thought to provide immunosuppressive tumor microenvironment (TME) and thereby support tumor growth. Glioma penetrates and actively interacts with the normal brain cells of its microenvironment, including various populations of neurons, astrocytes, and oligodendrocytes. The impact and contribution of glioma microenvironment to tumor growth and progression is a topic of extensive, albeit still young field of research. It is, therefore, becoming increasingly clear that along with the cell autonomous glioma proliferation, growth, and invasion, tumor-promoting activities of other cells of the microenvironment, as well as the communication lines between glioma and TME cells could be therapeutically targeted. Finally, a special, critical cell population for targeting is glioma stem cells. This rare and thus far only a partly defined type of glioma-initiating cells is considered as the most therapy-resistant, and largely responsible for GBM recurrence.

**Fig. 1:**
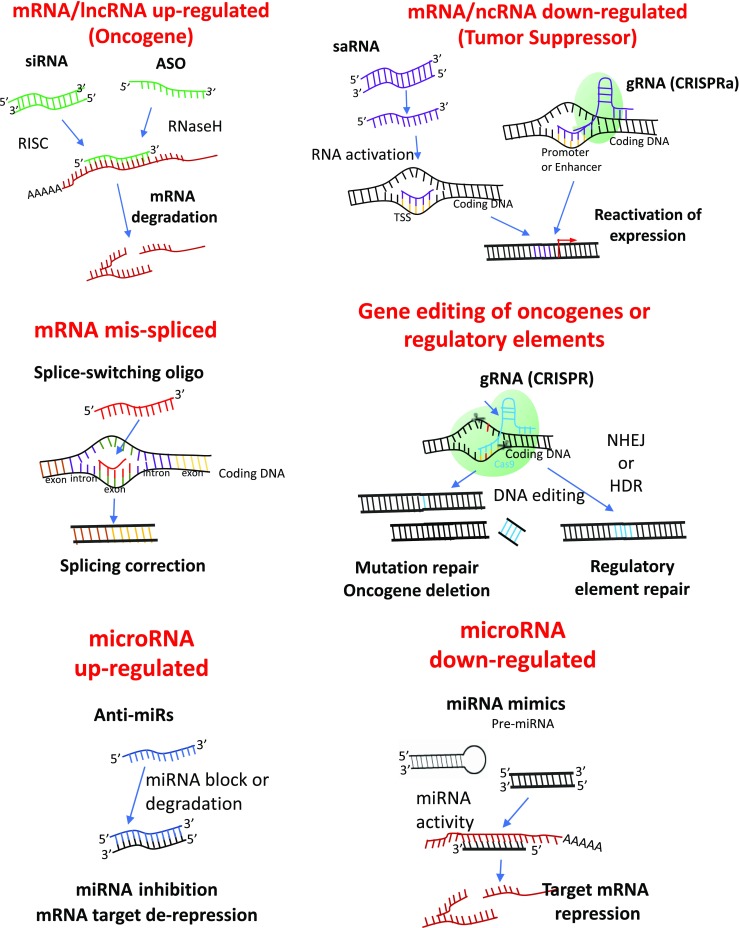
Oligonucleotide therapeutics for GBM

Although this review focuses on the top signaling pathways and putative molecular targets suitable for OT, it should be noted that tissue and cell-type–specific delivery and productive uptake of oligonucleotides have been thus far achieved for a few cell types only (e.g., hepatocytes) and that efficient targeting of selected cell populations in the brain tumors and CNS will require substantial advances of the field. Whereas drug delivery to the CNS is especially challenging because of the blood–brain barrier (BBB), high-grade gliomas and particularly GBM have partially disrupted BBB. Although this may be expected to facilitate delivery, previous trials have failed to demonstrate such an effect, perhaps owing to a significant delay between tumor cell infiltration and BBB disruption [4]. Therefore, development and optimization of the efficacious oligonucleotide drugs for malignant brain tumors sets a unique set of challenges but also presents opportunities for the field of oligonucleotide therapeutics (OT). In the sections below, we will overview the genetic, epigenetic, and expression landscape of GBM as it suggests the principles for prioritizing the oligonucleotide therapies. Of note, there are numerous genetic, epigenetic, and expression abnormalities found in GBM; we will describe only the most common and characteristic among them that could be potentially leveraged for the oligonucleotide targeting (i.e., “actionable” targets). Based on this information, we will propose the examples of putative OT that could be utilized for the 1) normalization/inhibition of the key overexpressed mRNA, miRNA, and other regulatory RNA species; 2) correction of splicing aberrations; and 3) editing driving mutations in protein-coding genes and regulatory parts of the genome (Fig. 1).

### Heterogeneity Defined by Mutations and Expression Patterns

A major hallmark of GBM and one of the reasons for its therapy resistant nature is the high intratumoral and intertumoral heterogeneity. Several classification systems have been developed to diagnose and stratify the GBM, and ultimately devise rational anti-GBM therapies. The Cancer Genome Atlas (TCGA), established for GBM more than 10 years ago, and for low-grade gliomas (LGG) more recently, integrated multidimensional genomic and expression datasets and, thus, largely contributed to current understanding of glioma etiology and heterogeneity [5, 6]. Adult GBM may arise *de novo* and present as primary GBM, or alternatively progress from lower-grade IDH-mutant glioma to so-called secondary GBM [7]. Morphologically, primary and secondary GBMs are largely undistinguishable; however, their genetics, molecular biology, clinical presentation, and prognosis are highly distinct. The majority of GBM cases (> 92%) manifest at advanced age (mean, 62 years) as the primary disease and are characterized by widespread anatomic distribution. Secondary GBM usually develops in younger patients (mean, 45 years); involves the frontal lobe, in particular the region surrounding the rostral extension of the lateral ventricles; and has significantly longer overall survival than primary GBM [8]. The major genetic marker of primary *versus* secondary GBM is the status of IDH1, the gene encoding isocitrate dehydrogenase 1, which is almost uniformly WT in primary GBM while mutated in secondary disease [8]. IDH1 mutations are also frequent (> 80%) in diffuse gliomas and a subset of anaplastic astrocytomas (WHO grades II and III, correspondingly), the precursor lesions of secondary GBM, as well as in oligodendroglial tumors of WHO grades II and III [9–11]. Although rare, IDH2 mutations are also observed in anaplastic oligodendrogliomas and oligoastrocytomas [12]. Therefore, IDH1/2 mutations could be considered as an early event in gliomagenesis, and they are preserved during progression to higher-grade disease.

The oncogenic effect of IDH mutations is thought to be at least twofold. The IDH enzymes catalyze the oxidative decarboxylation of isocitrate to α-ketoglutarate (α-KG). *IDH1/2* mutations are gain-of-function mutations that divert the enzyme to produce the oncometabolite 2-HG. Moreover, the catalytic rate is greatly increased, up to 100-fold, resulting in very high concentrations of 2-HG. Because of structural similarity, 2-HG inhibits enzymes that normally bind α-KG (either at the active site or an allosteric regulatory site), including HIF-1α resulting in upregulation of VEGF [13], as well as histone demethylases (e.g., prolyl hydroxylases, collagen prolyl-4-hydroxylase, and the ten-eleven translocation (TET) family of DNA hydroxylases [14], which in turn results in aberrant histone methylation. Changes in histone methylation impair cell differentiation and thus predispose to malignant transformation [15]. Finally, IDH1/2^mut^ display concerted CpG island hypermethylation at a large number of loci (G-CIMP phenotype), and this phenotype is associated with extended GBM survival. Conversely, the absence of *IDH* mutations and G-CIMP-low phenotype in LGG mark a distinct subgroup characterized by poor, GBM-like prognosis [6, 16]. Altogether, *IDH1/2*^*mut*^/secondary GBMs comprise a small and fairly homogeneous subclass of GBMs, whereas primary GBMs are more diverse.

The integrated analysis of TCGA further refined the GBM classification based on genetic and expression signatures reminiscent of different neural lineages. The initial analysis identified mesenchymal, classical, proneural, and neural expression patterns [17]. This classification has been recently revised to include only three major classes of classical, mesenchymal, and proneural gliomas that are broadly characterized by alterations in EGFR, NF1, and PDGFRA pathways, respectively. The neural GBM group was removed from this classification as likely reflecting the contamination by the normal neural cells [18]. Classical GBM is marked by the high-level EGFR amplification and a corresponding four-fold increase in EGFR expression, along with a significant proportion of the vIII EGFR mutation. In addition, neural precursor and stem cell marker NES, as well as Notch (NOTCH3, JAG1, and LFNG) and Sonic hedgehog (SMO, GAS1, and GLI2) signaling pathways, is highly expressed in this subtype [17]. Mesenchymal GBM is characterized by deletions, mutations, and the correspondingly reduced expression of NF1, coinciding in some cases with PTEN mutations. These events mark the activation of the Ras pathway and intersect with the Akt survival axis. The combination of higher activity of mesenchymal markers (CHI3L1 and MET) along with *CD44* and *MERTK* is reminiscent of an epithelial-to-mesenchymal transition that has been linked to dedifferentiated and transdifferentiated tumors [19]. Genes in the tumor necrosis factor super family pathway and NF-κB pathway, such as TRADD, RELB, and TNFRSF1A, are highly expressed in this subtype, potentially as a consequence of higher overall necrosis and associated inflammatory infiltrates in the mesenchymal class. The proneural GBM is featured by either IDH1 mutations or alteration of PDGFRA, including amplifications and mutations, and overall has a better prognosis. Proneural tumors with no PDGFRA aberrations are often mutated in PIK3CA*/*PIK3R1. Other genetic alterations that significantly impact gene expression, such as CDKN2A deletions and TP53 mutations, are more common to two or all three GBM subgroups. Overall, despite high genetic and expression heterogeneity of GBM, the TCGA analysis indicates that three major signaling pathways associated with cell cycle, senescence, and apoptosis are commonly dysregulated: RTKs/Ras/PI3K (in 88% of GBM), MDM/P53/CDKN1A/CDKN2A/apoptosis (87%), and CDKN2A/CDKs/RB1 (78%) [6, 16]. We will focus on the central targets for the OT, involved in these signaling pathways, in the section “mRNA Normalization by OT: Expanding the Repertoire of Targetable Molecules to Transcription Factors, RNA-Binding Proteins, and Other Protein Factors.”

In addition to the alterations in protein-coding sequences, several mutations in regulatory regions of the genome are strongly associated with GBM and might provide targets for the OT-guided function–restoration therapy. The most prominent among them are mutations in the telomerase reverse transcriptase promoter (*TERTp*) that have been reported in 80 to 90% of GBM [20, 21], at much higher rate than in other cancers. Normal brain tissues, with the exception of neural stem cells, do not express telomerase; however, in GBM, TERTp mutations (TPMs) lead to upregulation of TERT mRNA expression through the creation of a *de novo* transcription factor-binding site [22]. Generally, reactivation of telomerase activity is considered as a single most consistent feature of cancer. Essential for neoplastic growth, telomere lengthening and maintenance is required to escape replicative senescence. Telomerase may thus represent the most effective cancer therapeutic target [23]. Indeed, imetelstat, a competitive telomerase inhibitor, demonstrated promise in preclinical GBM models [24] and in the phase II study of pediatric brain tumors [25]. Curiously, the TCGA analysis indicates that TPMs correlate with generally reduced, rather than increased telomere length in GBM [20]. In contrast, mutations in the telomere-binding protein alpha thalassemia/mental retardation syndrome X-linked ATRX, which are nearly exclusive with the *TERTp* mutations, correlated with increased telomerase length and may thus underlie a telomere maintenance mechanism in GBM [26]. Although there are alternative mechanisms of TERT function proposed [23], and the whole spectrum of downstream consequences of TPM and TERT activation remains to be further investigated, correcting these hallmarks of the GBM with OT could represent a viable and robust approach.

### Epigenetic Alterations: DNA and Histone Modifications and 3D Organization

Extensive epigenetic remodeling that includes global DNA hypomethylation and chromatin remodeling takes place during gliomagenesis (reviewed in Gusyatiner and Hegi [27]). In addition to the critical function of IDH1/2 mutations in the DNA methylation patterns and epigenetic regulation described above, several epigenetic mechanisms have been implicated in gliomagenesis. The epigenetic silencing of the DNA repair gene MGMT by promoter methylation (pMGMTmet) in approximately half of GBM tumors is highly predictive for positive response to TMZ. The MGMT gene encodes the DNA repair enzyme O6-methylguanine-DNA methyltransferase that restores O6-methylguanine, the main adduct generated by TMZ, thereby blunting the effect of chemotherapy. Methylation status of pMGMT is often associated with the G-CIMP phenotype and currently used as a primary biomarker for risk stratification in gliomas (WHO 2016). Several studies validated a significant concordance between unmethylated pMGMT and MGMT protein expression and association of this status with reduced survival [28, 29]. These data suggest that lowering MGMT levels in patients with unmethylated pMGMT/high MGMT may sensitize them to TMZ therapy.

On a broader scale, TCGA-based studies continue to identify new alterations in the genes involved in chromatin organization. Although this topic has been thoroughly reviewed recently [27], several findings deserve attention as directly related to major epigenetic aberrations that are potentially targetable by OT. Generally, histone marks define the chromatin structure and, thus, the transcriptional activation status of a gene. These marks are established and regulated by numerous epigenetic modifiers, writers, readers, and erasers. The most common marks associated with active genes are trimethylation of lysine 4 (H3K4me3) and acetylation of lysines 9 and 27 (H3K9ac and H3K27ac), whereas inactive genes are usually marked with H3K9me3 and H3K27me3. Notably, in pluripotent and multipotent cells such as embryonic stem (ES) and neuroprogenitor cells (NPCs), critical regulatory genes may display both H3K4 and H3K27 trimethylation, a chromatin signature referred to as ‘bivalent domains’ that are poised for rapid activation or, diversely, silencing. Such events may shift a balance in expression of oncogenes and tumor suppressors, and lead to malignant transformation and tumorigenesis [30]. A major factor in this regulation PRC2 has histone methyltransferase activity and silences gene expression by dimethylating or trimethylating H3K27. One of its enzymatic subunits, enhancer of zeste homolog 2 (EZH2) has been reported as oncogenic in high-grade glioma (HGG) [31–33]. Histone acetylation is balanced by activities of histone acetyltransferases (HATs) and deacetylases (HDACs).

At least one of a set of 36 genes involved in chromatin and histone modifications is frequently altered in gliomas, most of which belonged to the IDH1/2 mutant-non-1p/19q-codeleted group [20]. Predicted glioma drivers associated with chromatin organization include ATRX, SETD2, ARID2, DNMT3A, SMARCA4, and ARID1A. ATRX, the most commonly mutated among them (in 37% of diffuse gliomas), forms a complex with DAXX and histone 3 variant H3.3, the genes frequently mutated in pediatric gliomas [34]. The ATRX–DAXX–H3.3 complex is associated with the alternative lengthening of telomeres. Also, the genes for histones themselves are frequently mutated in pediatric gliomas (will be discussed below). Interestingly, pediatric and adult GBM converge on dysregulation of H3.3 through mutations in the former and epigenetic repression via histone-lysine methyltransferase mixed lineage leukemia 5 (MLL5) in the latter. In adult GBM, MLL (which is upregulated in some cases) alters global chromatin conformation and suppresses differentiation of GBM stem cells [35].

Besides DNA methylation and histone modifications, 3D architecture of the chromatin emerges as an additional high-order regulatory layer controlling gene expression. This structural organization is largely mediated by the CCCTC-binding factor (CTCF) and cohesin complex that participate in the formation of highly conserved topologically associating domains (TADs). The majority of known promoter–enhancer interactions do not cross TAD boundaries. Disruption of such boundaries can promote differential gene expression in gliomas via creation of new promoter–enhancer pairs [36]. CTCF, whose activities are required in early development to regulate the balance between proliferation, differentiation, and survival of cortical progenitor cells [37], has been also directly implicated in gliomagenesis [36]. Specifically, IDH1 mutant gliomas exhibit hypermethylation at cohesin and CTCF-binding sites, thereby inhibiting the CTCF binding that is crucial for proper organization of TADs. Loss of the CTCF binding was shown to activate the enhancer of PDGFRA, a prominent glioma oncogene [36]. The full impact of CTCF and other TAD organizers on the dysregulated gene expression in gliomas remains to be investigated. Of note, a subset of gliomas showed mutations or copy number alterations in multiple genes involved in the cohesin complex, including the gene *STAG2* [20, 38]*.* Remarkably, a recent CRISPR-based screen demonstrated that mutations in CTCF and histone modifiers EP300 (HAT) and MLLs are among the most frequent events driving the astrocyte transformation to GBM in mice *in vivo* [39]. Altogether, this data suggests that targeting epigenetic modifiers and regulators of chromosomal topology may provide a feasible therapeutic approach for the GBM.

Of note, several drugs directed against epigenetic pathways (e.g., HDACs, mutant IDH1, EZH2, and DNMT) have been clinically tested for different malignancies, including glioma [27, 40, 41]. These strategies, in essence, aim to reverse dysregulated gene expression. However, because of the fundamental roles of the targets in both tumor and normal cells, they may lead to global effects on the genome and thus cause further dysregulation rather than normalization of expression. For example, inhibitors of DNA methylation showed preclinical efficacy *in vitro* and *in vivo* in IDH mutant glioma, and novel second-generation hypomethylating drug guadecitabine with improved pharmacodynamic characteristics is currently tested in a phase III study in AML. However, it remains controversial whether broad-spectrum demethylation is desired in glioma, as unwanted proto-oncogenes may be activated. Demethylation of the MGMT promoter may increase temozolomide resistance. Specific OT-based gene-targeted strategies, coupled with selective cell uptake and precise dosing regimens, could provide improved selectivity and specificity of targeting cancer cells.

### Pediatric Glioma

Although the most common glioma in adults is GBM, in children, low-grade gliomas and embryonal tumors, such as cerebellar pilocytic astrocytoma, pediatric diffuse astrocytoma, pleomorphic xanthoastrocytomas, and medulloblastoma, are the most prevalent. Although pediatric HGG is relatively rare, the prognosis is poor even with best available therapy. Histone mutations are very characteristic, such as the K27M mutation of the histone H3.1 or H3.3 isoform and G34R or G34V mutations of H3.3. The H3K27M mutation effectively turns on gene expression by not allowing trimethylation of the H3 Lysine 27 residue for transcriptional silencing [42]. Although the H3K27M mutation does not provide a direct therapeutic target, downstream effectors such as specific transcription factors (TFs) may be identified as suitable targets. Pediatric high-grade gliomas also commonly harbor IDH1 R132H and BRAF V600E mutations, as well as CDKN2A, NF1, TP53, PDGFRA, PIK3CA, PIK3R1, and FGFR1 alterations and NTRK fusions [43, 44], providing further potential therapeutic targets.

Ultimately, mutations and epigenetic alterations that accumulate during gliomagenesis lead to a profoundly dysregulated gene expression, which manifests at the levels of mRNAs/proteins, microRNAs, and other regulatory RNAs. Prior to discussing candidate RNA targets for the OT, we provide a brief overview of the oligonucleotides as the CNS therapeutics with the focus on drugs tested in human clinical trials for GBM.

## Development of Oligonucleotide Therapeutics for Brain Tumors

### Clinical Experience and Considerations

Oligonucleotides are short, DNA- or RNA-based synthetic polymers (usually 13-30 nucleotide long) that are considered as a gene-modulatory class of drugs, along with small molecules and antibodies. Oligonucleotides bind to RNA (and in some cases to DNA) through Watson–Crick base pairing and, upon binding, modulate functions of the targeted nucleic acids in a sequence-specific fashion. Therefore, OT can be developed to target both protein-coding and regulatory nonprotein-coding RNA (ncRNA), as well as protein-coding and regulatory DNA sequences of the genome with high specificity. Traditionally, the major classes of oligonucleotides developed for therapeutics were single-stranded ASOs that promote mRNA target degradation by engaging RNase H cleavage or modulate mRNA splicing via steric block of specific splice sites and exon skipping. Overall, in the past 5 years, over 100 antisense OTs have been tested in phase I clinical trials, a quarter of which have reached phase II/III. More recently, the field expanded with siRNAs and miRNA-modulatory oligonucleotides (both antagonists and mimics) and is currently further spurred with the emergence of oligonucleotide-guided gene-editing technologies such as CRISPR/Cas9 (Fig. 1).

Unmodified DNA and RNA oligonucleotides are inherently unstable; to become useful as drugs, they must be chemically modified to increase their nuclease resistance, but also retain or enhance tissue distribution, cell uptake, and target recognition [45–47]. The predominant cell uptake mechanism for oligonucleotides is endocytosis, and the majority of internalized oligonucleotides are trapped in the endocytic compartment leading to poor target recognition and low functional activity. Systemic administration results in rapid and broad tissue distribution within just a few hours, with the highest concentrations accumulating in the liver and kidney. Both size and charge of most oligonucleotides prevent their distribution across the intact BBB. Nevertheless, modified oligonucleotides administered by intrathecal injection distribute broadly in the CNS. Recent advancements that enable OT delivery to the brain and malignant gliomas via local (intratumoral, intrathecal, convection-enhanced delivery (CED)) and systemic (intravenous, intranasal) routes are reviewed in the section “Oligonucleotide Delivery to Malignant Glioma: Selective Targeting with Aptamers, Cell-Penetrating Peptides, and Nanoparticles.”

The fairly limited clinical attempts to investigate oligonucleotide drugs for various neurologic diseases have been previously reviewed [45–47]. Importantly, the OT approach was approved by FDA for spinal muscular atrophy (SMA), a single-gene inherited neurological condition, and clinical trials on amyotrophic lateral sclerosis (ALS) and Huntington’s disease (HD) are ongoing [48, 49]. However, brain tumors such as GBM might present considerable additional challenges. For the treatment of SMA, caused by homozygous deletion of the SMN1 gene, intrathecal administration of a modified ASO nusinersen increases the amount of the SMN protein in alpha motor neurons by altering the distribution of alternatively spliced transcripts from the SMN2 gene [50]. In this case, relatively low levels of the SMN protein (10-20% of wild type) may be sufficient for the normal functioning of alpha motor neurons. Furthermore, even a subset of motor neurons that can be preserved or restored to normal function can sustain mobility and respiration. Accordingly, nusinersen was shown to produce clinically significant improvements in treated infants [51, 52]. However, the required modulatory effects of oligonucleotides employed in GBM treatment are likely to be substantially higher, because virtually all tumor cells need to be reached and eliminated, in order to have a therapeutic effect. Small molecule drugs, such as receptor tyrosine kinase inhibitors (RTKi) tested thus far, showed no benefit for GBM, although effective in other malignancies such as lung or kidney cancer. Oligonucleotides are typically much larger than small molecule drugs (393.4 g/mol for the EGFR inhibitor erlotinib and 7127.2 g/mol for nusinersen); this not only makes administering equivalent molar quantities of the OT more difficult but also reduces BBB penetrance and tissue distribution of the drug. Thus, OTs will only be effective if these pitfalls are circumvented, as outlined in the sections below.

Four reported human clinical trials tested OTs for GBM thus far. The first phase II trial, concluded in 2005 [53], used aprinocarsen, a phosphorothioate ASO to the 3′-untranslated region of human PKC-α mRNA, that inhibited PKC-α expression through RNase H-mediated cleavage of the mRNA [54]. This trial was based on the early work from 1987 to 1999 that suggested the benefits of PKC-α inhibition in several animal models of cancer, but included only minimal preclinical data relevant to glioma and limited to U87 model only [55]. Aprinocarsen was administered at 2 mg/kg/day to patients with HGG as a continuous intravenous infusion in 21/28-day cycles. Neither neurologic toxicity nor tumor responses or clinical benefits were observed. The effect of PKC-α inhibition on the BBB integrity was suspected, and no follow-up studies were reported.

Another set of trials tested a transforming growth factor-β2 inhibitor trabedersen, a phosphorothioate ASO, in recurrent high-grade gliomas [56–58]. TGF-β is a potent cytokine with multiple biological activities which became attractive for GBM targeting because of its role in glioma proliferation, migration, invasion, angiogenesis, and immunosuppressive properties [59]. The safety and efficacy of trabedersen have been established through pharmacokinetic and toxicology studies *in vitro* and in rabbits and primates *in vivo* [60]. In phase I/II dose escalation studies on adult HGG patients, the maximum tolerated dose was not reached [57], indicating a favorable safety profile of the oligonucleotide. A follow-up randomized and controlled phase IIb study of 145 patients with recurrent or refractory AA and GBM further evaluated the efficacy and safety of 2 doses (10 and 80 μM) administered intratumorally by CED. A benefit of 10 μM trabedersen *versus* standard chemotherapy for the 14-month tumor control rate and a trend for its superiority for 2-year survival rate of AA (but not GBM) patients was reported [56]. However, the study was criticized for suboptimal design and insufficient statistical power [61, 62]. Additional multinational phase III study designed to test the efficacy and safety of 10 μM trabedersen was discontinued in 2014 because of slow recruitment. Despite the disappointing lack of conclusions of these trials, they validated the safety of the locally administered phosphorothioate oligonucleotides for glioma patients. More recent preclinical data further reinforced TGF-β as a relevant target in GBM growth and radioresistance [63, 64].

A set of clinical trials utilized the immunostimulating oligodeoxynucleotides containing unmethylated cytosine-guanosine motifs (CpG-ODN). Phase I and II trials with the CpG-ODN infused into surgical cavity after GBM removal concluded that, although well tolerated at doses up to 20 mg, the drug did not improve survival [65, 66]. Notably, these past trials utilized early generation of OTs and have examined neither drug delivery and distribution in the brain tumor tissues nor target engagement or specific biomarker response. Therefore, the lack of efficacy can be attributed to poor delivery, lack of activity, or suboptimal selection of the molecular target.

Imetelstat (GRN163L, Geron Corporation, Menlo Park, CA), a covalently lipidated 13-mer-thiophosphoramidate oligonucleotide that binds to the template region of the RNA component of telomerase, and thus serves as competitive telomerase inhibitor, demonstrated promise in preclinical GBM models [24] and was tested in the phase II study of recurrent pediatric brain tumors [25]. The drug was administered as intravenous 2-h infusion at 285 mg/m^2^ to patients with HGG, DIPG, medulloblastoma, and ependymoma. Although the regimen proved too toxic in children and the trial was terminated, it was concluded that the drug crossed the BBB and achieved both intratumoral and PBMC target inhibition [25].

**Fig. 2:**
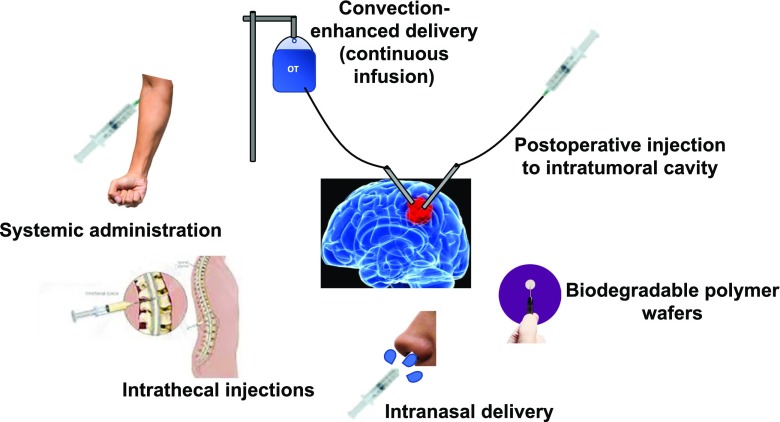
Strategies for OT administration to malignant glioma

This summary indicates a critical need in a thorough strategy for the identification of the most promising molecular targets for OT for HGG, development of the specific glioma-targeting OT, and carefully designed preclinical and clinical studies to test their distribution, efficacy, safety, and target engagement in HGG. Potential strategies for OT administration to HGG are illustrated in Fig. 2.

### Characteristics and Limitations of Current Preclinical Cell and Animal Models

Preclinical studies of candidate therapeutics for malignant gliomas have long suffered from poor animal models and often relied on cultured glioma cell lines only (reviewed in McNeill et al. [67]). For decades, glioma drug discovery has been largely carried out on a few cell lines, with U87 being the most commonly used among them for practical rather than biological reasons. These lines, grown in fetal serum, exhibit molecular characteristics that are vastly different from primary glioma cells, weakly tumorigenic, noninvasive, and scantly vascularized in nude mice, and thus not reliably modeling human GBM. In contrary to human HGG, these orthotopic models also have largely intact BBB, further limiting their relevance for glioma drug discovery programs. Newer models based on xenografts derived from low-passage glioma-initiating cells (GSCs) cultured in defined serum-free conditions or passaged in mice *in vivo* more faithfully recapitulate the infiltrating character of human GBM and are more suitable for drug discovery and optimization studies, including these on OTs. However, such models exhibit higher interexperimental variability and are, thus, more labor-intensive and costly. It should be also noted that subcutaneous models of glioma lack the natural brain microenvironment comprised of multiple nonglioma cell types contributing to the intratumoral communication and tumor growth [68], and thus represent an inadequate surrogate of the disease. In addition to glioma xenografts and allografts, genetically engineered mouse models (GEMs) bearing characteristic mutations and specific alterations in signaling pathways have been developed and provide a benefit of the immunocompetent brain TME [67]. Altogether, with increased recognition of the molecular heterogeneity of HGG ecosystems, employment of multiple complementary *in vitro* and *in vivo* models should become a standard requirement for glioma drug development, to ultimately increase the success of clinical trials.

### Oligonucleotide Delivery to Malignant Glioma: Selective Targeting with Aptamers, Cell-Penetrating Peptides, and Nanoparticles

Recent work suggests that chemically stabilized oligonucleotides can be delivered to orthotopic infiltrating GBM in mice via various delivery routes (intratumoral injections, CED infusions, and systemic intravenous and subcutaneous injections), both unformulated and formulated in LNPs, and lead to functional inhibition of a target and significant effects on tumor growth [69–71]. However, further improvements in chemistry are needed to increase the oligonucleotide stability and half-life, glioma-selective uptake, endosomal escape, potency, and target specificity. It should be also noted that in mice, BBB penetrance, delivery, and distribution of a given oligonucleotide in the tumor tissues, and thus its overall efficacy, depend largely on a GBM model, its cellular properties, invasiveness, and vascularization.

The OTs composed of only of DNA or RNA bases are rapidly degraded in a biological system by endo- and exonucleases and phosphatases. The efforts of the field are focused on the development of 1) sugar, backbone, and end modifications improving OT nuclease resistance and stability; 2) tail modifications enhancing tissue uptake and distribution; 3) chemical conjugations to various targeting moieties (e.g., aptamers, cell-penetrating peptides, and antibodies); and 4) formulations with lipid, magnetic, and other nanocarriers to further enhance the selective targeting and internalization of the oligonucleotides (reviewed by Geary et al. [47], Khvorova and Watts [72], and Lima et al. [73]). The contemporary, clinically advanced OTs are partly or fully stabilized molecules with modified backbones, with natural phosphodiester bonds replaced by phosphorothioate (PS), phosphodithionate (PS2), phosphonoacetate (PACE), phosphorodiamidate morpholino oligomers (PMO), or peptide nucleic acid (PNA). Most commonly used sugar modifications include 2′-OMe, 2′-O-MOE, LNA, 2′-F, ethylene-bridged nucleic acid (ENA), BNA, and UNA. Combinations of various sugar modifications with the PS backbone are commonly utilized. The comprehensive overviews of the oligonucleotide evolution history have been recently published [72, 74, 75]; here we will focus on the progress in advancing the oligonucleotide designed to target malignant gliomas.

Several approaches based on the receptor-mediated uptake have been developed to enhance the efficiency and selectivity of oligonucleotide targeting to glioma cells and tumors. One of them utilizes aptamers, or “chemical antibodies,” short single-stranded nucleic acids (RNA or ssDNA) selected from a partially random oligonucleotide library via the *in vitro* procedure denominated systematic evolution of ligands by exponential enrichment (SELEX) [76]. Molecules bound with high affinity and specificity to a cell surface, complex macromolecular structures, or receptor of interest can be identified in multiple rounds of positive and negative selection and further optimized. Similar to antibodies, aptamers have a broad range of applications such as therapeutics and imaging agents. The first aptamer to reach phase II clinical trials for several cancers, AS1411, is a nucleolin-specific oligonucleotide. Nucleolin is a ubiquitously expressed cell surface marker for various cancer cells; therefore, although initially developed for nonglioma cells [77], AS1411 also binds to glioma. AS1411 appears to inhibit the proliferation of glioma cells but not normal astrocytes *in vitro* and prolong the survival of subcutaneous U87 glioma-bearing mice [78]. Nanoconjugates of chemotherapeutics such as paclitaxel with AS1411 showed enhanced glioma cytotoxicity compared with paclitaxel without aptamer [79]. Additional studies identified the aptamers that bind to glioma EGFRvIII, PDGFRb, EphB2/3, and tenascin-C receptors and examined their conjugation with several siRNAs, miRNAs, and anti-miRNAs (reviewed by Delac et al. [76] and Amero et al. [80]). For example, the PDGFRβ-targeted aptamer chimera bearing the siRNA to STAT3 reduced glioma cell viability, tumor growth, and angiogenesis in a subcutaneous model of glioma [81]. The major flaw of many prior SELEX screens, however, is that they utilized one or few glioma cell lines only (most often U87), and thus, the selected aptamers may not be efficacious for heterogeneous glioma cells and tumors. More systematic approaches for the identification of ligands and aptamers targeting a variety of tumor cells, including tumor-initiating stem cells in multiple heterogeneous tumors, still have to be developed [82]. Although the utility of aptamer conjugates with siRNA and other OT for orthotopic GBM remains to be explored, recent pilot findings denoting that aptamers may cross the BBB and accumulate in the GBM [83, 84] and suggest their potential as a new type of therapeutic vehicles.

An alternative approach to improve the internalization of OT is through the small nontoxic cell-penetrating peptides (CPP) that utilize endocytosis and direct translocation but may also internalize via the receptor-mediated uptake (e.g., via EGFR [85]) and thus provide selectivity for tumor cells such as GBM. Although the CPPs appear fairly efficient as transfection reagents to glioma cells *in vitro* [86, 87], only limited *in vivo* data have been reported thus far. In one study, conjugation of the stathmin-targeted siRNAs to a PP75 CPP appeared to improve the effect of siRNA when the complex was injected to subcutaneous U251 glioma xenografts [88]. A rationally designed amphipathic α-helical peptide NF55 produced stable nanoparticles with nucleic acids, promoted endosomal escape, and demonstrated efficacy in several animal models, including an orthotopic GBM model [89]. A recent report described the first example of an antibody-antisense strategy directed to GSCs. The GSC-targeting CD44 antibody was conjugated with a stabilized ASO targeting the gene downregulated in renal cell carcinoma (DRR), a genetic driver of GSC invasion [90], and mediated the internalization of the ASO and DRR knockdown [91]. Additional conjugates of the oligonucleotides, for example with neuroactive lipids such as cholesterol, have also been shown to significantly improve their distribution and uptake in the orthotopic GBM [69].

The BBB presents the major challenge for systemic drug administration to all neurologic diseases. However, in malignant brain tumors, the BBB is partially disrupted and leaky, potentially enabling a facilitated route for a drug to the tumor, compared to the brain parenchyma. Nevertheless, the degree of the BBB penetrance varies among the tumors and may diminish when a successful therapy reduces the tumor burden, making the subsequent treatments of the residual tumor less efficacious. Approaches to further improve drug delivery through the BBB and monitor the quantitative uptake at different stages of tumor growth and recurrence are, thus, warranted. Recent clinical trials suggested that repeated opening of the BBB with a pulsed ultrasound, in combination with systemic microbubble injections, is well tolerated in GBM patients and has the potential to facilitate chemotherapy [92] and delivery of other drugs such as OTs [93]. Limited work has been carried out to investigate and improve the BBB penetrance of the OT for brain tumors thus far. Several CNS-targeting ligands such as Rabies virus glycoprotein (RVG) peptide [94, 95], apolipoprotein E [96–98], Angiopep-2 [99], and transferrin [100] were proposed to improve drug delivery to the brain parenchyma through the BBB and may provide a starting point for the development of the ligand/receptor-mediated targeting to HGG too. Further work in the field should capitalize on these discoveries.

Several formulations of OTs in nanoparticles (NPs), including lipid and metal (e.g., gold or iron) NPs, have been investigated in preclinical glioma studies. NPs may provide carriers that would increase the stability and reduce renal clearance of the OT and other systemically administered drugs (reviewed in Lozada-Delgado et al. [101]). They can be decorated with ligands such as a glioma-specific peptide chlorotoxin [102] to potentiate the selective targeting. Only a few of the preclinical studies led thus far to human clinical trials on GBM, such as the ongoing phase II trial of the cationic liposomes encapsulating the cDNA for wild-type p53 [103]. The first human safety trial administering intravenously a siRNA utilizes spherical nucleic acid nanoparticles (SNAs) and is currently recruiting GBM patients [104]. The SNAs consist of gold nanoparticles (13 nm AuNPs) covalently bound with densely packed, highly oriented siRNA duplexes to oncoprotein Bcl2L12 [105]. The particles can cross the BBB through targeting class A scavenger receptors [106], and they are anticipated to reduce the Bcl2L12 expression and thus sensitize glioma toward therapy-induced apoptosis by enhancing effector caspase and p53 activity.

Finally, the natural nanoparticles—cell-derived extracellular vesicles (EVs) or exosomes—have recently attracted researchers as potential vehicles for the OT delivery to various cells and tissues. As EVs contain endogenous small nucleic acids, efficiently internalized by various recipient cells [68, 107] and can be loaded with synthetic oligonucleotides [108], they may provide an alternative targeting strategy. The efficacy and safety of this new delivery approach, along with the optimal cell sources for the production of EV therapeutics, remain to be investigated.

***Box 1. Major advantages of the oligonucleotide therapies***

**Table:** 

*- Once the biological and chemical principles defining the ON delivery, distribution, and efficacy in the brain and brain tumors and overall low toxicity are established, they are expected to be adaptable for multiple targets in combination therapies. Relative to antibody and small molecules, OT drug development is faster, cheaper and does not involve large-scale target screens, design, and production efforts.* *- OT technology substantially expands the repertoire of targetable molecules, as the protein factors previously considered nontargetable (e.g., TFs and RBPs) can be modulated at the level of mRNA transcripts, along with regulatory RNA such as miRNAs and lncRNAs.* *- Potential applications of OT include transcription activation by small activating RNA and oligonucleotide-guided genome editing.* ***Overall, OT may offer potential advantages of high specificity and selectivity, low toxicity, and possibility of combination therapies.***	

## mRNA Normalization by OT: Expanding the Repertoire of Targetable Molecules to Transcription Factors, RNA-Binding Proteins, and Other Protein Factors

Initially, OTs have been proposed for the modulation of mRNA levels and splicing. Apparent mRNA targets for the GBM combination therapies can be devised from previous attempts to utilize small molecule drugs targeting the RTKs, which have been successful in other cancers but failed as monotherapies in GBM. Gefitinib, erlotinib, afatinib, and osimertinib are first-line treatment choices for patients with locally advanced or metastatic non-small cell lung cancer harboring epidermal growth factor receptor tyrosine kinase mutations [109]. Sunitinib, pazopanib, and temsirolimus are among the first-line treatment options for metastatic renal cell carcinoma [110]. The reasons for RTKi trial failures in GBM are thought to be diverse and include poor penetration of the drug into the tumor tissue, lack of reliance of the GBM cells on one particular signaling pathway, and rapid and effective metabolism of the drug by tumor cells [4]. For example, EGFR blockage may be circumvented by GBM cells by PDGFRA, ERBB2, or MET pathways [5]. Upstream activation of the EGFR pathway by NFKBIA deletion is common in GBM [111].

### OT Is Uniquely Selective Allowing TF Targeting

Oligonucleotides may offer distinct advantages for targeting TFs, a feat that is especially difficult to achieve with small molecule inhibitors [112]. Candidate upregulated transcription factors include ZEB1, YAP/TAZ, MRTF-A, Gli-1, CREB, ETS-1, STAT3, TWIST1, HOXC10, EZH2, BMI1, JMJD6, P53, ATF5, REST, SP1, and NFAT [112–128], whereas others such as KLF6 are downregulated [129]. ELK4 downregulation was shown to reduce Mcl-1, an anti-apoptotic protein in GBM, resulting in reduced tumor formation in xenograft models. Transcription elongation factors were also proposed as potential GBM targets as discovered in a siRNA screen [123].

TP53 (p53), a DNA-binding protein and regulator of cell cycle arrest and apoptosis, is an obvious candidate as it is commonly mutated in GBM, often with the acquisition of oncogenic features. In fact, TP53 is the single most frequently mutated gene in GBM [17], with overall mutation frequency of about 30%. The mutations are characteristically rare in the classical subtype; however, their rate exceeds 50% in the mesenchymal subtype. The mutations are often not only loss-of-function but turning TP53 from tumor suppressor to an oncogene in a dominant-negative fashion. Mutant TP53 typically accumulates to very high levels in GBM, contributing to malignant progression [130, 131]. Targeting TP53, especially the highly expressed dominant-negative form is difficult with small molecule drugs [132]. TP53 reactivation is being attempted with zinc metallochaperone-1, as well as drugs such as APR-246, PK11007, PK7088, and COTI-2. Despite its advantages to target oncogenic TP53, no OT has yet been proposed to our knowledge.

It is presently unclear which transcription factors are the most promising targets, and indeed, many may provide initial treatment response, although a few considerations appear important to overcome GBM recurrence [133]. Tumor heterogeneity is likely to account for treatment failure, both in terms of resistance to chemotherapy and disease recurrence [134]. Understanding expression patterns relative to morphological features will aid the development of therapies that can overcome the inherent resistance of GBM arising from its heterogeneity [133]. GSCs are identified based on their ability of tumor initiation and recognized as more treatment resistant [135]. Highly mobile invading tumor cells are also more likely to survive local treatment including surgery and radiation and initiate recurrence. Finally, random mutations and expression alterations in tumor cells give rise to evolving subpopulations that provide diversity with the potential treatment-resistant tumor cell fractions. These subpopulations are initially colocalized within the tumor and subsequently increase relative to others and migrate to distant sites [136]. Tumor cells are hypothesized to communicate via intercellular microtubes that enhance treatment resistance and invasion [137]. Taken together, successful strategies will effectively target GSCs as well as the highly mobile tumor cells.

### Preclinical Studies to Target GSCs by OT

GSCs are reported to have activated POU3F2, SALL2, SOX2, and OLIG2, all four simultaneously, and in combination, and the inhibition of each one leads to the reversible loss of tumorigenicity [138]. In addition to these presumed driver transcription factors, other signaling pathways are also activated, such as Wnt/β-catenin, Notch, PI3K, NF-κB, SHH/GLI, COUP-TFII, and JAK/STAT/stathmin [139–144]. Members of the STAT family of transcription factors are of particular interest as key regulators of cell growth, angiogenesis, motility, and immune response [145]. STAT3 is reported to be a strong oncogenic driver of GBM, and CRISPR/Cas9-mediated deletion of STAT3 in a GBM-derived cell line blocked tumorigenesis by specifically eliminating GSCs [116, 146]. Perivascular GSCs are reported to exhibit a proneural expression pattern and have activated EZH2, whereas GSCs in hypoxic regions feature a mesenchymal phenotype and express BMI1 protein. Using genetic or pharmacologic inhibition of both, all GSCs in the tumor were effectively inhibited leading to improved survival in a mouse model [120]. The zinc finger-containing transcription factor Gli-1 is the main transcriptional mediator of the Sonic Hedgehog (SHH) signaling pathway and is highly expressed GBM. siRNA-mediated downregulation of Gli-1 expression resulted in reduced proliferation and increased apoptosis *in vitro* [147]. MEF/ELF4 was found to be highly expressed in GBM, the level of expression correlated with poor prognosis as well as stem-like cellular characteristics [148]. PRMT5 is a methyltransferase and putative splicing regulator that affects GSC self-renewal. Its expression is also associated with more aggressive disease [149]. Silencing of PRMT5 expression in GBM-derived cell lines results in apoptosis and reduces tumorigeneity *in vivo* [150]. Knockdown of the ecto-nucleotidase ENPP1 in cultured GSCs induced differentiation, cell cycle arrest, and apoptosis [151].

### OT to Modify DNA Methylation and Histone Modification

Epigenetic regulators have a key role in GBM development demonstrated by the IDH-mutant disease. ING5 has been shown to enhance self-renewal and tumor formation. Such epigenetic regulators are potential targets for treatment [152]. TLX knockdown by siRNA inhibited human GBM tumor formation in mice via upregulation of TET3 expression [153]. AJAP1, a cell junction protein, was found to be deleted or epigenetically silenced in most GBMs, and restoration of expression resulted in decreased tumor cell migration [154]. ARNT2, a HIF family member, is proposed a possible GBM target as the regulator of GBM aggressiveness via histone methylation [155].

### Key Aspects of the GBM Tumor Microenvironment Emerge as OT Targets

Emerging data supports the role of the GBM TME both as enabling disease development and actively supporting GBM cells in progressive disease. GBM TME contains a large number of non-GBM cells, such as macrophages, microglia, myeloid cells, lymphocytes, astrocytes, and endothelial cells. The GBM TME is highly immunosuppressive and actively enhancing GBM growth. Macrophages and microglia appear to constitute substantially to the tumor mass [156]. Inhibition of colony-stimulating factor-1 receptor (CSF-1R) by BLZ945 was shown to block glioma progression [157]. Inhibition of CSF-1R by PLX3397 was reported to reduce tumor cell proliferation and glioma progression in a PDGF-B-driven proneural glioma mouse model, although having no direct effect on cultured GBM cells. In contrast, the receptor tyrosine kinase inhibitors dovitinib and vatalanib were effective to reduce GBM cell growth *in vivo*, but had no anti-tumor effects *in vivo* [158]. Although the mouse model used in the study rapidly develops resistance to CSF-1R inhibition, combining IGF-1R or PI3K blockade with CSF-1R inhibition delayed resistance and increased survival [156]. Natural killer cells infiltrating the tumor are reported to inhibit GBM growth via secreted TNF-α in response to PDGF-DD, an isoform of PDGF, which in turn, is produced by GBM cells. This intrinsic break in GBM growth can be enhanced by CpG oligonucleotide treatment [159]. Recent evidence suggests that GBM-associated endothelial cells contribute to resistance to VEGF pathway inhibitors by downregulating VEGFR-2, and dual inhibition of VEGFR and PDGFR may overcome this resistance [160]. The collagenase genes MMP1 and MMP13 are overexpressed in 5% of GBM specimens and promote tumor invasion [161]. NF1 deficiency of GBM is associated with increased tumor-associated macrophages and microglia infiltration [18].

### Alternative OT Targets: Splice Regulation, RNA Processing, Combination Therapy to Attack Multiple Signaling Pathways, and Overcoming TMZ Resistance

Splice-switching oligonucleotides (SSOs) have also been applied to GBM treatment. Of the alternatively spliced Mnk2a/Mnk2b products, Mnk2a is a tumor suppressor, whereas MnK2b is oncogenic. In U87MG cells, an SSO effectively increased Mnk2a expression although normally the cells express Mnk2b almost exclusively. This led to reduced colony formation in soft agar [162]. A similar approach was applied to Bcl-xL/Bcl-xS splice-switching. Bcl-xL, an anti-apoptotic protein, is preferentially expressed in GBM, whereas the alternatively spliced Bcl-xS version is pro-apoptotic and highly efficient to produce programmed cell death. An SSO was tested in commonly used GBM cell lines *in vitro* [163]. Targeting polypyrimidine tract-binding protein 1 (PTB1) offers a distinct approach to target mRNA splicing and selective modify protein isoforms [164].

Although specific signaling pathway activations are found in subsets of GBM [165], inhibition of such signaling pathways thus far showed disappointing results [166]. Continued efforts are needed to look for effective targets. For example, combination oligonucleotide therapy has the promise of effective growth suppression through pathway inhibition to avoid the emergence of resistant clones although exhibiting tolerable toxicity [167–169]. A test of a target-pair combination approach to block key signal transduction pathways showed that simultaneous knockdown of CK2α and EGFR/EGFRvIII resulted in downstream target deactivation, reduced GSC marker expression, and prolonged survival in a mouse model [170].

A number of other potential GBM treatment targets are being explored. MGMT is of great interest as the mediator of TMZ resistance of GBM [171, 172]. Targeted depletion of KPNB1, a nuclear protein transport receptor, resulted in efficient upregulation of pro-apoptotic Bcl-2 family members, resulting GBM cell death [173]. There is evidence that neuronal activity may promote GBM growth via neuroligin-3 secretion, offering further potential therapeutic targets [174]. Dopamine receptor D4 antagonists have been identified in a drug screen to selectively kill GBM cells [175], suggesting that elements of the dopamine pathway may be effective targets. RBM14 controls nonhomologous end-joining (NHEJ) DNA repair and knockdown of RBM14 sensitizes glioma spheroids to radiation [176].

RNA processing and specific RNA-binding proteins (RBPs) appear to be significantly altered in GBM and are of interest as potential targets [70, 177]. RBPs are regulators of RNA splicing, capping, polyadenylation, transport, decay, localization, and translation. The polypyrimidine tract-binding protein PTBP1 was found to be highly expressed in many GBM specimens, causing aberrant splicing of annexin A7, a tumor suppressor, in turn resulting in enhanced EGFR signaling. PTBP1 expression is low in glial cells and derepressed in GBM as a result of deregulated miR-124 or PTBP1 amplification [178]. Splicing factor hnRNPH is also frequently upregulated in GBM. As a result, the death-domain adaptor protein Insuloma-Glucagonoma protein 20 undergoes erroneous splicing to generate an anti-apoptotic variant, causing absent TNF-α/TRAIL apoptosis signaling [179]. DDX1, an RNA helicase, is overexpressed in a subset of GBM specimens [161]. The conserved RNA-binding protein Musashi1 is proposed as a major regulator of splicing in GBM cells, affecting cell adhesion, migration, and invasion [180, 181]. Alternative polyadenylation of mRNA transcripts plays a key role in carcinogenesis and was shown to occur in GBM [182]. Better understanding of the mechanism may lead to the identification of potential GBM vulnerabilities. Knockdown of SNRPB, a component of the splicing machinery in a GBM cell line, resulted in apoptosis [183].

Finally, tumor suppressor (TS) genes play an important role in GBM development. CDKN2A is homozygously deleted in 52%, PTEN in 36%, p53 in 35%, and NF1 in 18% of GBM samples. Restoration of TS expression is being tested for various tumor types [184] and may be applied to GBM. Such an approach, however, would have to overcome several obstacles; a large number of TSs are involved, and some of them have a wide range of inactivating mutations; therefore, therapy must be individualized. Furthermore, restoration of expression needs to be near-universal, or rapid tumor progression would occur. Presently, such barriers to TS reactivations seem insurmountable; however, emerging gene-editing technologies may advance this strategy.

Table 1 presents a summary of select candidate genes for GBM OT that are the most promising to lead to trial candidates, based on the prevalence of alterations and preclinical evidence of efficacy. Prior OT clinical trials conducted for malignancies other than GBM should be both encouraging to the potential of this approach to target disease but also sobering to the task ahead (Table 2). The apparent efficacy to treat lymphoma but not solid tumors highlights the crucial importance of delivery. Experience from these efforts will no doubt help to find an effective OT implementation for GBM.

**Table 1:** Candidate mRNAs and protein-coding genes for inhibition therapies in GBM

mRNA	Dysregulation	Pathway	
EGFR	Amplification, mutation	RTK signaling	[185–187]
TERT	Transcriptional activation	Telomere lengthening	[188, 189]
STAT3	Upregulated	Cytokine signaling	[81, 190, 191]
Gli-1	Upregulated	Hedgehog signaling	[147, 192]
Notch1	Upregulated	Notch signaling	[193, 194]
Myc	Upregulated	Mitogenic signaling	[195–197]
Wnt	Activated	Wingless signaling	[198–200]
PI3K	Activated	Signal transduction	[201, 202]
NF-κB	Activated	Cytokine production	[168, 203]
EZH2	Activated, upregulated	Self-renewal	[33, 120]
POU3F2	Activated	Self-renewal	[204–206]
SALL2	Activated	Self-renewal	[204–206]
SOX2	Activated	Self-renewal	[204–206]
OLIG2	Activated	Self-renewal	[204–206]

Targets that appear the most promising to lead to OT-based trials are selected based on the prevalence of alterations and preclinical evidence of feasibility and efficacy

**Table 2:** OTs in oncology clinical trials: select agents that may be considered for repurposing to GBM

Drug	Target	Disease	Result/reference
Phase III			
Oblimersen/G3139	Bcl-2	Advanced melanoma	Negative [207]
		CLL	Positive [208]
		Multiple myeloma	Negative [209]
Custirsen/OGX-011	Clusterin	Prostate cancer	Negative [210, 211]
Phase II			
AZD9150	STAT3	Malignant ascites	Unpublished
IONIS-STAT3Rx	STAT3	Advanced cancers	Unpublished
Veglin	VEGF	Mesothelioma	Sponsor withdraw support
DCR-MYC	Myc	Hepatocellular carcinoma	Unpublished
Apatorsen/OGX-427	Hsp-27	Prostate cancer	PFS unchanged but biomarker improved [212]
		Urothelial carcinoma	Nonsignificant improvement in OS [213]
		Urothelial carcinoma	Negative [214]
		Pancreatic cancer	Nonsignificant improvement in PFS/OS [215]
LErafAON	c-Raf	Head and neck cancer	Unpublished
		Clear cell renal cell cancer	Unpublished
IMO-2055	TLR9	Clear cell renal carcinoma	Unpublished
EMD 1201081	TLR9	Head and neck cancer	Negative [216]
AEG35156	XIAP	Hepatocellular carcinoma	Positive [217]
		Acute leukemia	Negative [218]
		Breast/pancreas/NSCLC	Unpublished
Phase I			
MTL-CEBPA	C/EBP-α	Hepatic carcinoma	Unpublished
PNT2258/PNT100	Bcl-2	Advanced solid cancers	Mild lymphocyte and platelet count drop [219]
EZN-2968	HIF-1α	Advanced solid tumors/lymphoma	Unpublished
LErafAON	c-Raf		Safe and well tolerated [220]
AZD4785	KRAS	Advanced solid tumors	Unpublished
ISIS 183750	eIF4E	Advanced solid tumors	Safe and well tolerated [221]

PFS = progression-free survival; OS = overall survival

## MicroRNA Normalization by OT

### Complexity of MiRNA Regulation and Challenges in Identification of Most Efficacious Therapeutic MiRNA for OT Targeting

MiRNAs are small RNA regulators of various cellular processes that are heavily implicated in cancer and among the most promising targets for the OTs [222, 223]. MiRNAs control gene expression post-transcriptionally, by binding to partly complementary sites in mRNA targets, with sometimes only 6-8 nucleotide binding sufficient for mRNA destabilization and translational repression. Each miRNA can regulate the levels of numerous targets, including the genes that either promote or counteract carcinogenesis. Therefore, miRNAs themselves can exhibit either tumor-promoting and tumor-suppressive properties and were shown to contribute to the development of many cancers. Deregulation of miRNA expression has been associated with cancer initiation, progression, and metastasis; consequently, miRNAs have been proposed as both biomarkers and therapeutic targets for various malignancies [222, 224]. Distinct miRNA signatures also characterize gliomas of high and low grades, and thus, miRNAs present excellent biomarkers for these diseases [225, 226]. Furthermore, miRNAs regulate all aspects of glioma growth including tumor cell proliferation, invasion, survival, angiogenesis, cancer stem cell properties, immune escape, and therapy resistance (reviewed in Zhang et al. [227]). Many miRNAs have been associated with HGG, and numerous mRNA targets validated to different extent for each of them. It should be noted that because of the complexity of the miRNA regulation, there is a trend in the field to reduce miRNA functions to one or a few selected targets, the approach that rarely reflects the true nature of miRNA regulation. Furthermore, a validated mRNA target in one cell type may not be regulated by the same miRNA in other cell types. Such diversity of miRNA regulation has been observed not only among the cells of different cancers, but also heterogeneous glioma cell lines [228–230]. Consequently, functions of specific miRNAs are often context and cell type dependent and dictated by the balance and stoichiometry of multiple co-expressed targets. For example, miR-17-92 is an established polycistronic oncogene [231], and its inhibition leads to decreased proliferation of glioma spheres *in vitro* [232]. However, the higher rather than the lower levels of miR-17 are strongly associated with extended survival of GBM patients [71, 233], probably because of its alternative functions in other cells of the GBM microenvironment [234]. Conversely, an established tumor suppressor miR-34a, whose mimic has reached phase I clinical trials for treating select cancers [235], appears as a “risk factor” in GBM [71, 233] and, thus, may not be a therapeutically relevant for this disease. Finally, modulation of some miRNAs, such as miR-148 and miR-26a, may be beneficial for subsets of glioma in a subtype-specific manner [71, 236, 237].

These examples highlight the challenges of identifying the most efficacious therapeutic miRNA candidates for inhibition or supplementation. Although numerous miRNAs have been functionally associated with GBM (reviewed by Zhang et al. [227]), most of them require rigorous follow-up analyses preceding any clinical development. In the following sections, we will focus on several best validated miRNA targets for OT, including tumor-promoting miRNAs that could be inhibited by anti-miRs and tumor-suppressing miRNAs for potential oligonucleotide replacement therapies. Generally, miRNA inhibitors are single-stranded ASO, bearing modifications similar to those utilized in ASO for mRNA targeting. They reduce miRNA levels or activity via RNaseH mechanism or interfere with miRNA incorporation to RICS. Typically, miRNA mimic oligonucleotides are double stranded, principally similar in their design to siRNAs; however, the single-strand design seems also feasible [238]. In this section, we will also list the best validated mRNA targets that mediate miRNA activity in GBM and may provide functional readouts for the efficacy of miRNA modulation by the anti-miRs and mimics.

### Key MiRNAs for Inhibition Therapies

MiR-21 was the first miRNA discovered that was upregulated in GBM [239] and is the most widely studied tumor-promoting miRNA in various cancers. We and others have provided detailed reviews of its expression and cancer-associated functions previously [240, 241]. MiR-21 is encoded in a plastic, frequently activated chromosome 17q23 locus, and its high expression is a characteristic feature of multiple malignancies. In GBM, it has been implicated in tumor cell cycle, survival, invasion, angiogenesis, and therapy resistance via numerous direct targets and downstream signaling pathways [241]. Our early studies have demonstrated that miR-21 regulates more than 500 mRNAs in glioma cells and suggested at least 70 of them as direct targets containing miR-21-binding sites [242]. Among the validated direct targets are the tumor suppressor PDCD4, metalloprotease inhibitors RECK and TIMP3 involved in invasiveness and angiogenesis, a molecular core of the apoptosome APAF1, the cell cycle regulator CDC25A, the transcriptional inhibitor NFIB, a negative regulator of Ras signaling Spry1/2, and insulin-like growth factor (IGF)-binding protein-3 [243, 244]. PTEN and STAT3 have been suggested as additional miR-21 targets, but this is still controversial [245, 246]. Involvement of several key targets in miR-21 tumor-promoting functions has been demonstrated in mouse models *in vivo* [242, 243, 247]. Altogether, these data provide a strong rationale for miR-21 clinical targeting, alone or in combination with other drugs. Of note, however, miR-21 is highly expressed in various cells and tissues and contributes to immune response and other normal functions [241, 248], and the consequences of its local intracranial or systemic inhibition have yet to be assessed.

In addition to cultured glioma cells [87, 249], several studies attempted miR-21 inhibition in established intracranial GBM, using various antagomiRs and delivery techniques. For example, injections of the miR-21 antisense-ODN complex with amphiphilic R3V6 peptide suppressed tumor growth of C6 glioma [250]. A selective small molecule inhibitor of miR-21 maturation (AC1MMYR2), when injected intraperitoneally to mice bearing orthotopic U87 tumors inhibited tumorigenesis [251]. An interesting recent study suggested that selective brain tumor targeting can be achieved with three-way-junction (3WJ)-based RNA nanoparticles, artificially derived from pRNA of bacteriophage phi29 DNA packaging motor. Repeated systemic treatment with the folate-conjugated 3WJ RNP harboring anti-miR-21 8-mer LNA sequence (FA-3WJ-LNA-miR21) targeted orthotopic GBM and reduced tumor growth in a PDX mouse model [252].

The miRNA miR-10b appears to be a very promising miRNA target for malignant gliomas. This miRNA is not expressed in the healthy brain cortex, whereas its high levels appear as the common marker of the malignant state, including both primary and metastatic brain malignancy [253]. Although undetectable in normal glia, neuron, and neuroprogenitor cells, miR-10b gets transcriptionally activated in most gliomas of both low and high grades [70, 253]. Moreover, it appears to elicit tumor “addiction,” in that there seems to be no alternative pathway to bypass miR-10b loss [254]. MiR-10b is essential for glioma viability and its inhibition by ASO or deletion by gene editing is detrimental for the tumor but has no obvious negative effects on normal neural cells [253, 254]. The downstream effects of miR-10b expression involve glioma cell cycle, invasion, and resistance to apoptosis [70, 229, 230, 253, 255–258]. Among its targets are cell cycle inhibitors CDKN1A/p21 and CDKN2A/p16, pro-apoptotic gene BCL2L11/Bim, and MBNL splicing factors [70, 253]. HOXD10, initially also reported as a direct miR-10b target, remains controversial [253, 259]. Notably, despite several validated miR-10b targets, the mechanism of glioma addiction to miR-10b remains largely unknown and involves unconventional mechanisms [70]. A number of formulations and carriers have been tested for anti-miR-10b delivery to cultured glioma cells, including the PLGA nanoparticles [260] and PDGFR-targeted aptamers [84]. Various formulations of miR-10b ASO and delivery routes for orthotopic GBM were explored [70]. This included intratumor injections of lipophilic formulations, convection-enhanced delivery of the cationic LNPs via osmotic pump, and systemic delivery of unformulated stabilized PS 2′-O-MOE anti-miR-10b oligonucleotide. Notably, both local and systemic administration of anti-miR-10b in intracranial glioma models led to the derepression of mRNA targets and slowed down tumor growth. Furthermore, high doses of systemically administered miR-10b inhibitors (up to 150 mg/kg) appear safe in mice [70]. This data encourages further development of both local and systemic miR-10b-targeting therapies.

In addition, our recent work also demonstrates the feasibility of gene-editing strategies for brain tumors *in vivo* and suggests that CRISPR/Cas9-medited miR-10b gene ablation may provide a new and highly effective therapeutic approach that directly eliminates the key oncogenic dependency of gliomas [254]. Importantly, virus-mediated gene editing of the miR-10b locus is much more efficient in glioma than in normal neuroglial cells that do not express this miRNA. This result suggests a wide therapeutic window for the miR-10b-editing systems targeting malignant brain tumors.

The closely related miR-221 and miR-222 paralogue miRNAs are frequently upregulated in gliomas and characterized as an oncogenic cluster. High levels of these miRNAs appear as a strong risk factor associated with shortened survival including both low- and high-grade glioma [71, 261]. Based on TCGA analysis, miR-222 expression correlates with proliferation, anti-apoptosis, and cell migration-related bioterms in gliomas [71]; it promotes glioma cell invasion and confers therapy resistance [261, 262]. Combined inhibition of the two miRNAs with PNAs conjugated with an octa-arginine tail promotes apoptosis and sensitized glioma cell lines to TMZ [263]. MiR-221/222 target the cell growth suppressive cyclin-dependent kinase inhibitors CDKN1B/p27^Kip1^ and CDKN1C/p57 ^Kip2^ and thereby enable bypassing quiescence [264, 265], along with the mediator of apoptosis PUMA [266]. Other targets associated with the regulation of glioma invasion include the protein tyrosine phosphatase μ (PTPμ) [267] and connexin 43, a tumor suppressor and major component for the establishment of gap junction intercellular communication in glial cells, which is frequently reduced or deleted in high-grade gliomas [268].

Therapeutic inhibition of this cluster has been proposed for various cancers, including prostate, melanoma, hepatocellular carcinoma, and multiple myeloma [269–272]. Intratumor injections of its LNA ASO reduced the growth of subcutaneous U251 glioma [261]. However, to the best of our knowledge, the effects of miR-221/222 inhibition in orthotopic GBM have not yet been investigated. While assessing the therapeutic potential of this approach, future studies should also consider the consequences of miR-221/222 inhibition on the regulation of a putative target MGMT and overall health of normal brain tissues expressing relatively high levels of miR-221/222.

### Key MiRNAs for Replacement Therapies

Although miRNA upregulation in GBM considered is an active process and most consistently upregulated miRNAs exhibit tumor-promoting functions, reduced levels of specific miRNAs in tumor cells can be viewed merely as a consequence of the lost cellular identity. Indeed, the miRNA signature of neuronal differentiation is lost in glioma, and the miRNAs implicated in the differentiation of neuroglial lineages are those downregulated in the disease. Therefore, replacement of such molecules may potentially lead to the reduced cycling of tumor cells and differentiation of glioma stem cells. In addition, several miRNAs have been characterized as tumor suppressors that regulate important oncogenic proteins. It should be emphasized that development of potent miRNA mimics for GBM treatments is likely to be even more challenging than miRNA inhibitors, because of the impact of chemical modifications required for efficient tissue distribution, and the productive uptake and stability of the OT on the miRNA activity in target regulation are currently unknown.

One of the most prominent candidates for replacement in gliomas is miR-124, an established neurogenic regulator highly enriched in brain neurons and the miRNA most strongly downregulated in HGGs ([273, 274] and our unpublished data). MiR-124 is generally one of the best investigated ncRNAs, because of its very high brain-specific expression. Its fundamental role in neuronal differentiation, alongside miR-9/9^∗^, includes the control of chromatin accessibility, DNA methylation, mRNA expression, and alternative splicing to induce a default neuronal state [275–278]. Its loss in glioma is associated with upregulation of numerous validated targets critical for glioma proliferation, stemness, migration, and radio- and chemoresistance. Some of the key miR-124 targets are listed in Table 3. Of note, miR-124 expression exhibits inverse correlation with many of its targets in TCGA glioma specimens, suggesting that, indeed, this miRNA is their prime regulator whose loss in GBM results in the massive derepression of the protumorigenic proteins (our unpublished data).

**Table 3:** Top candidate miRNAs for inhibition or replacement therapies

MicroRNAs dysregulated	Regulated process	Major validated direct mRNA targets in glioma	References
MiRNA for inhibition therapies			
MiR-10b	Cell cycle, cell death, invasiveness	CDKN1A, CDKN2A, BCL2L11/BIM, TFAP2C, MBNL1-3, APAF	[70, 253, 255, 279]
MiR-21	Migration, invasion, apoptosis, angiogenesis	PDCD4, RECK, TIMP3, APAF, CDC25, NFIB, Spry2, IGFBP3	[242–244, 280]
MiR-221/222	Cell cycle, apoptosis, invasiveness	CDKN1B/p27^Kip1^, CDKN1C/p57 ^Kip2^, PUMA, PTPμ, Cx43	[264–266, 268]
MiR-148a	Stemness, angiogenesis, apoptosis	MIG6, BCL2L11/BIM, FIH1, QKI, SKP1, GADD45	[71, 236, 281, 282]
MiRNA for replacement therapies			
MiR-124	Neurodifferentiation, stemness, proliferation, migration	CDK6, CDK4, NRAS, PIM3, PTBP1, TEAD1, ROCK1, LAMB1, IQGAP1, LAMC1, ITGB1	[178, 283–289]
MiR-128	GSC renewal, proliferation, angiogenesis	BMI1, SUZ12, E2F3a, p70S6K1	[290, 297, 298, 387]
MiR-7	Proliferation, angiogenesis, drug sensitivity	EGFR, FAK, IRS-1, IRS-2	[301–304]
MiR-181	Proliferation, apoptosis, invasiveness, drug sensitivity	MGMT, K-RAS, BCL-2, KLF6	[306–308]
MiR-218	Invasiveness, apoptosis, chemosensitivity, angiogenesis	EGFR, PLCγ1, PIK3C2A, ARAF, ECOP, LEF1	[299, 300, 388]
MiR-137	Proliferation, neurodifferentiation, invasion	CDK6, COX-2, PTVP-1, YBX1, CDC42, c-KIT	[84, 274, 389–391]

MiR-124 is one of a few master regulators that not only promotes the neurodifferentiation of normal and glioma stem cells [274, 278] but also has a capacity to reprogram human fibroblasts to functional neurons [275, 291]. Its supplementation reduces the ability of tumor cells to survive under oxygen and nutrient deprivation [283]. In addition, miR-124 enhances T-cell effector cytokine production via STAT3 signaling and thereby reverses glioma-mediated immunosuppression [292]. Therefore, both local and systemic administration of miR-124 mimics seem a promising therapeutic for glioma treatments. The efficacy of systemically injected miR-124 encapsulated in bilipid nanoparticles or overexpressed in exosomes has been tested in murine models of GBM and suggested survival benefit [293, 294]. In addition, miR-124 delivery to orthotopic U87 tumors was attempted by intratumor injections of the miR-124-overexpressing mesenchymal stem cells [295, 296]. The therapeutic efficacy of these approaches remains to be established.

MiR-128 is also highly expressed in neurons although it is lost in the early stages of gliomagenesis [290], with promise as a replacement therapy. Inhibiting the glioma cell cycle, GSCs renewal and radioresistance, miR-128 targets epigenetic regulators BMI1 and SUZ12, the key components of PRC1 and PRC2, respectively [290, 297]. It also suppresses mammalian target of rapamycin (mTOR) target p70S6K1 and its downstream signaling molecules such as HIF-1α and VEGF and, thus, attenuates tumor growth and angiogenesis [298].

Other strong candidates for replacement therapies include miR-7, miR-137, miR-181d, and miR-218 [84, 274, 299, 300]. For example, miR-7, a miRNA characterized as tumor suppressor for multiple cancers, regulates glioma growth and invasions via targeting EGFR, focal adhesion kinase, and IRS-1, IRS-2, and other genes, and downstream Akt pathway [301, 302]. It modulates angiogenesis, and its systemic administration in biodegradable nanoparticles targeting both EC and tumor cells reduces angiogenesis and tumor proliferation in glioblastoma xenografts [303]. MiR-7 was also identified as a potent sensitizer for TRAIL-induced apoptosis in GBM cells [304]. miR-181d, whose reduced expression is strongly associated with poor GBM survival [71], was proposed as a tumor suppressor for gliomas. It regulates the expression of critical GBM-driving genes such as MGMT, K-Ras, and Bcl-2 [305, 306]. Other miR-181 family members may contribute to TMZ sensitivity of glioma [307] and increased permeability of the blood–tumor barrier by targeting transcription factor Kruppel-like factor 6 [308].

Of note, many additional miRNAs have been suggested as either tumor promoting or suppressive for gliomas (reviewed in Zhang et al. [227]), or contributing to chemo- and radioresistance of HGGs [309]. However, the data is often insufficient or inconsistent to move the candidates to a more advanced preclinical stage. For example, miR-182 appears protective against glioma based on the TCGA analysis [71, 310]; it targets oncogenes such as c-Met, HIF2A, and Bcl2L12, it sensitized glioma to therapy-induced apoptosis, and its intravenous administration in SNA nanoparticles reduces tumor burden and increases the survival of mice with the orthotopic GBM [310, 311]. However, the same miRNA has oncogenic and metastatic properties in multiple other cancers, including medulloblastoma [312–314]. It also triggers TGF-β1-dependent NF-κB induction in glioma cells and provokes a more aggressive glioma phenotype by targeting the deubuiquitinase CYLB and protocadherin-8 [315, 316]. Such results reflect the high complexity and context-dependent functions of miRNAs, pointing to important roles of the tumor microenvironment and the tumor subtype-specific genetic landscape in shaping miRNA responses. It also highlights the requirement for diverse in-depth preclinical studies utilizing heterogeneous GBM models and leading to miRNA-based personalized OT medicine. Table 3 lists select candidate miRNAs for GBM OT that are the most promising to lead to trial candidates, based on the prevalence of alterations and preclinical evidence of efficacy.

## LncRNA

The vast majority of the human genome is transcribed into various classes of ncRNAs, including diverse regulatory species, whereas mRNA accounts for only a small part of human transcriptome. Among the ncRNAs, in addition to miRNAs, are thousands of long noncoding RNA (lncRNA) species. These transcripts are longer than 200 nt and transcribed in sense or antisense orientation to protein-coding genes, or within intergenic regions. Many lncRNAs are expressed in a highly tissue-specific manner and dysregulated in cancer. Strikingly, 40% of human lncRNAs (equivalent to 4000-20,000 lncRNA genes) are expressed specifically in the brain [317], and many of them exhibit dynamic spatiotemporal patterns. However, only a small subset of them has been explored thus far, opening up an exciting new area for research and potential therapeutic interventions. Unlike miRNAs, lncRNAs may operate via multiple molecular mechanisms that include chromatin remodeling, transcriptional interference, miRNA and RBP sequestration, modulation of splicing and translation, protein scaffolding, and others [318]. It is, thus, important to consider subcellular distribution, compartmentalization, and molecular partners of lncRNAs for their targeting, as many (if not most) of them operate in the nuclear domain or protein complexes not easily accessible by traditional RNA-targeting machineries such as RNAi [318, 319]. Additional challenge in studying lncRNAs is associated with their characteristic species specificity that limits cellular and animal modeling of their functions. Significant efforts are required to better understand the biology and functional diversity of lncRNAs, improve and standardize lncRNA nomenclature and databases, and reveal their therapeutic potential.

Similarly, substantial efforts will be required to understanding the rules of the oligonucleotide-mediated lncRNA targeting and optimization of the corresponding OTs. Generally, a sequence-specific design of ASOs and siRNAs utilized for mRNA targeting has been also applied for the lncRNAs [320]. Thus, degradation of lncRNAs has been achieved via RNase H cleavage and RNAi machinery, correspondingly. As a remarkable example, application of combinatorial 2′-O-MOE DNA ASOs resulted in the efficient long-term knockdown of a long nuclear UBE3A antisense transcript (1000 kb) in cultured neurons and CNS of an Angelman syndrome mouse model [321]. LNA-, PNA-, and SNA-based OTs have been also proposed for targeting cytosolic and nuclear lncRNAs [322–324]. Of note, although RNAi is viewed as a largely cytosolic pathway, siRNAs have been also shown to target nuclear lncRNAs both transcriptionally and post-transcriptionally [325, 326]. Overall, although multiple reports led to the recognition of lncRNAs as compelling targets for OT, no study has yet systematically tackled the unique technical challenges associated with silencing lncRNAs, which are shorter, of lower abundance, more structured, and more likely to contain repeats than mRNA [327, 328]. Development of the therapeutic targeting of lncRNA will undoubtfully require detailed mechanistic studies of the individual lncRNA candidates and their interacting partners. It will also benefit from the research addressing off-target effects associated with targeting lncRNA and the development of new bioinformatics and computational tools enabling the assessment and prediction of such off-targets.

With the research of lncRNAs in glioma exponentially growing in the past 5 years, lncRNAs have been implicated in glioma growth, invasion, stem cell maintenance, angiogenesis, and BBB penetrance [329]. Nevertheless, it might be still premature to propose the candidate lncRNAs for therapeutic targeting; we will thus list only on a few lncRNAs that are upregulated in HGGs, associated with the disease progression and prognosis [330], and have been reported as oncogenic in several independent publications. The most studied among them is HOTAIR, a HOX transcript antisense RNA that promotes glioma cell cycle, growth, and angiogenesis [331, 332]. The mechanism of its function in glioma is not fully investigated and may include EZH2-dependent epigenetic regulation [333], as well as regulation of β-catenin signaling [334] and perturbed miRNA binding [335–337]. Another lncRNA H19 is processed from the H19-IGF2 loci, can be induced by c-myc and HIF1α, and is upregulated in glioma and other malignancies [338, 339]. It has been implicated in tumor invasion, angiogenesis, and resistance to TMZ [340–344]. MiRNA binding and sequestration by H19 has been proposed as the major mechanism underlying its functions; however, additional studies are required to further evaluate its role in gliomagenesis and therapeutic potential. CRNDE, a lncRNA initially discovered in colorectal cancer, also appears highly upregulated and associated with disease progression in gliomas [345]. The proposed mechanisms of CRNDE-induced glioma growth include miR-384 targeting and its downstream signaling [346], as well as inhibition of mTOR pathway via P70S6K phosphorylation [347]. LncRNA taurine upregulated 1 (TUG1) coordinately promotes GSC self-renewal by antagonizing miR-145 in the cytoplasm and recruiting polycomb via YY1 binding to repress differentiation genes in the nucleus [348]. It has also been associated with glioma angiogenesis and VEGFA expression [349]. Additional interesting, potentially glioma growth-promoting lncRNAs that require further exploration are NEAT1 [350, 351], XIST [352, 353], FOXM1-AS [354], CCAT1/2 [355, 356], EXONEXIN [357], and lncRNAs expressed from the HOXA locus [358, 359]. In addition, controversial evidence suggests both oncogenic and tumor-suppressive functions for specific highly abundant glioma lncRNAs such as the key invasion and metastasis-associated MALAT1 [360, 361], indicating a need in the careful follow-up investigations.

Notably, very little effort has thus far been reported to target lncRNA in the orthotopic glioma models. Practically all animal studies relied on the *ex vivo* manipulations to examine the effects of lncRNA on the growth of glioma tumors. In this venue, a notable experiment was reported by Katsushima et al., in which an ASO-targeting TUG1 delivered in cyclic Arg–Gly–Asp (cRGD) peptide-conjugated polymeric micelles was intravenously injected to mice bearing the orthotopic proneural GBM [348]. The treatment has been administered at 1 mg/kg per day over 4 weeks, twice a week, leading to the induction of GSC differentiation and efficient repression of tumor growth. Although the advancement of OT targeting the tumor-promoting lncRNA only recently emerged as an important new research avenue for exploration, it may require special considerations for the development of chemistries and formulations efficacious for this new class of therapeutic targets, governing pleiotropic functions in both nuclear and cytosolic compartments.

It is also worth noting the expression of numerous putative tumor suppressor lncRNAs in glioma (reviewed in Peng et al. [329]). Some of them have been implicated in tethering and, thereby, reducing functional activity of oncogenic miRNAs; for example, lncRNAs CASC2 and Gas5 mediate the repression of tumorigenic miR-21 and miR-222, correspondingly [362, 363]. Activation of such TS lncRNA should be considered as additional therapeutic approach, and oligonucleotides could be leveraged for the gene-editing strategies aimed at such activation, as outlined below.

## Immunomodulatory Cancer Therapy

Efforts to apply OT therapy to initiate antitumor immunity are overshadowed by the recent achievements with checkpoint inhibitor antibodies and T cells with engineered chimeric antigen receptor (CAR-T), in B-cell lymphoblastic leukemia [364], non-Hodgkin lymphoma [365], esophagogastric cancer [366], non-small cell lung cancer [367], melanoma [368], and renal cancer [369]. Despite these successes, the efficacy of checkpoint inhibitors [370] or CAR-T [371–375] in GBM has not been demonstrated thus far. There is preclinical data for EGFR and HER2 CAR-T therapy for pediatric brain tumors and clinical trials are being carried out [376]. Although CD19 CAR-T had shown strong efficacy in non-Hodgkin lymphoma and B-cell lymphoblastic leukemia, up to 40% of patients developed neurotoxicity of various severity, termed CAR-T cell-related encephalopathy syndrome (CRES) [364]. It is currently unclear if there is a therapeutic window for GBM CAR-T, or indeed, any immunostimulatory treatment given the presumably even higher risk of CRES. Nevertheless, testing of various ways to achieve antitumor immune response is being explored. MiR-17-92 expression in T cells enhances T-cell survival and interferon production [377]. CRISPR/Cas9-mediated knockout of DGK was found to improve antitumor T-cell activity [378]. As mentioned before, CpG oligonucleotide treatment of tumor-associated NK cells resulted in reduced tumor growth [159]. Stimulation of the innate immune receptors MDA5 or RIG-I with their natural ligands p(I:C) and 3pRNA also improved antitumor activity [379].

## Expanding the Universe: OT Guides for Genome Editing

The OT has a remarkable potential to broaden the space of potential targets for gliomas and other CNS disorders, to include mRNAs encoding diverse protein factors, along with various classes of regulatory RNAs. Moreover, and perhaps most exciting, is that in addition to the traditional RNA-targeting applications of the OTs (e.g., ASO, SSO, siRNAs, miRNA modulators), recent studies indicate that oligonucleotides may activate gene transcription [380] and provide advantageous guides for genome-editing technologies as well [381]. Plasmid and viral delivery systems for Cas9-sgRNA applications *in vivo* have multiple limitations associated with system design, efficacy, and durability of exposure to the editing machinery. In contrast, chemically synthesized oligonucleotide gRNAs could enhance genome editing activity by reducing susceptibility to nuclease degradation and at the same time minimizing the exposure to editing machinery and thus off-target effects and leading to improved therapeutic efficacy [381–385]. A recent study of lipid nanoparticle formulations of such gRNAs and mRNA encoding Cas9 has already demonstrated clinically relevant levels of genome editing *in vivo* [386], highlighting the therapeutic potential of this system. As genome editing may provide a unifying strategy for correcting mutations, deleting oncogenes, activating tumor suppressors, and potentially also editing regulatory elements and 3D genome architecture, its applications to human diseases are practically unlimited. Our recent work demonstrated both feasibility and efficacy of CRISPR–Cas9 gene editing (of miR-10b) in intracranial GBM models [254] and, thereby, opened up the area of therapeutic gene editing for malignant brain tumors. Future studies will investigate the applications of oligonucleotide-guided gene editing for correcting the key aberrations underlying gliomagenesis at the levels of genome and transcriptome. Correcting mutations (e.g., in EGFR, IDH, P53), modifying promoter marks (e.g., MGMT methylation), and editing genetic and epigenetic architecture to normalize gene expression, as well as deleting mRNA and ncRNA oncogenes at the genomic level, may provide alternative and complementary OT-based strategies to those targeting RNA, along with the more conventional therapies.

## Abbreviations

GBM: Glioblastoma
WHO: World Health Organization
LGG: Low-grade glioma
TMZ: Temozolomide
OT: Oligonucleotide therapeutics
TS: Tumor suppressor
BBB: Blood–brain barrier
IDH: Isocitrate dehydrogenase
2-HG: 2-Hydroxyglutarate
CNS: Central nervous system
CED: Convection-enhanced delivery
ASO: Antisense oligonucleotide
SSO: Splice-switching oligonucleotides
SMA: Spinal muscular atrophy
ALS: Amyotrophic lateral sclerosis
HD: Huntington’s disease
AA: Anaplastic astrocytoma
TME: Tumor microenvironment
GEM: Genetically engineered mouse
BNA: Bridged nucleic acid
UNA: Unlocked nucleic acid
LNA: Locked nucleic acid
EV: Extracellular vesicles
pRNA: Bacteriophage packaging motor RNA
ncRNA: Nonprotein-coding RNA
RBP: RNA-binding protein
PFS: Progression-free survival
OS: Overall survival
